# Epidemiological Features, Clinical Symptoms, and Environmental Risk Factors for Notifiable Japanese Encephalitis in Taiwan From 2008 to 2020: Retrospective Study

**DOI:** 10.2196/63053

**Published:** 2025-01-28

**Authors:** Fu-Huang Lin, Yu-Ching Chou, Chi-Jeng Hsieh, Chia-Peng Yu

**Affiliations:** 1 School of Public Health, National Defense Medical Center, Taipei City, Taiwan; 2 Department of Health Care Administration, Asia Eastern University of Science and Technology, New Taipei City, Taiwan

**Keywords:** epidemiology, Japanese encephalitis virus, domestic, environmental factor, retrospective study

## Abstract

**Background:**

Japanese encephalitis (JE) is a zoonotic parasitic disease caused by the Japanese encephalitis virus (JEV), and may cause fever, nausea, headache, or meningitis. It is currently unclear whether the epidemiological characteristics of the JEV have been affected by the extreme climatic conditions that have been observed in recent years.

**Objective:**

This study aimed to examine the epidemiological characteristics, trends, and potential risk factors of JE in Taiwan from 2008 to 2020. Specifically, the study focused on gender, age, season, residential area, clinical manifestations, high-risk areas, and the impact of environmental and climate factors.

**Methods:**

This study reviewed publicly available annual summary data on reported JE cases in the Taiwan Centers for Diseases Control between 2008 and 2020.

**Results:**

This study collected 309 confirmed domestic patients and 4 patients with imported JE. There was an increasing trend in the incidence of JE, 0.69‐1.57 cases per 1,000,000 people, peaking in 2018. Case fatality rate was 7.7% (24/313). Comparing sex, age, season, and place of residence, the incidence rate was highest in males, 40‐ to 59-year-old patients, summer, and the Eastern region, with 1.89, 3.27, 1.25, and 12.2 cases per million people, respectively. The average coverage rate of the JE vaccine for children in Taiwan is 94.9%. Additionally, the major clinical manifestations of the cases included fever, unconsciousness, headache, stiff necks, psychological symptoms, vomiting, and meningitis. The major occurrence places of JE included paddy fields, pig farms, pigeon farms, poultry farms, and ponds. For air pollution factors, linear regression analysis showed that SO_2_ (ppb) concentration was positively associated with JE cases (*β*=2.184, *P*=.02), but O_3_ (ppb) concentration was negatively associated with them (*β*=−0.157, *P*=.01). For climate factors, relative humidity (%) was positively associated with JE cases (*β*=.380, *P*=.02).

**Conclusions:**

This study is the first to report confirmed cases of JE from the surveillance data of the Taiwan Centers for Diseases Control between 2008 and 2020. It identified residence, season, and age as risk factors for JE in Taiwan. Air pollution and climatic factors also influenced the rise in JE cases. This study confirmed that JE remains a prevalent infectious disease in Taiwan, with its epidemic gradually increasing in severity. These findings empower clinicians and health care providers to make informed decisions, guiding their care and resource allocation for patients with JE, a disease that significantly impacts the health and well-being of the Taiwanese population.

## Introduction

The Japanese encephalitis virus (JEV) is a significant public health concern in Asia, causing an estimated 68,000 clinical cases annually, primarily in East Asia and Southeast Asia [1]. Although symptomatic Japanese encephalitis (JE) occurs infrequently, it has a serious case fatality rate, with up to 30% of encephalitis cases resulting in death. Encephalitis survivors have a high likelihood of developing permanent neurological or psychiatric sequelae, with an estimated occurrence rate of 30%-50% of cases. The endemic transmission of JEV poses a health threat to over 3 billion people in a substantial portion of the World Health Organization Southeast Asia and Western Pacific regions [1]. The disease currently lacks specific antiviral treatment, necessitating supportive care measures like managing symptoms and providing respiratory and nutritional support to help patients overcome the infection [2]. Vaccination stands as the effective strategy to prevent the disease, with World Health Organization recommending the integration of JE vaccination into national immunization programs in all regions where the disease poses a significant public health threat [1,3].

JE is a mosquito-borne viral infection caused by the JEV, a member of the *Flaviviridae* family closely related to dengue, yellow fever, and West Nile viruses [4]. The primary means of transmission of JEV from animals to humans is through the bite of infected mosquitoes, particularly Culex species, with *Culex tritaeniorhynchus* (*Cx. tritaeniorhynchus*) being of particular importance. Pigs and ardeid birds serve as the main amplifying hosts for the virus [5]. The clinical presentation of JE in humans varies widely, encompassing a spectrum ranging from mild or asymptomatic infection to severe encephalitis. This severe form of the disease is characterized by significant mortality, with a high death rate among infected individuals. Additionally, survivors of severe JE often experience sequelae within the central nervous system [6].

In recent years, air pollution has been identified as a significant public health concern in China, with an estimated 1 million deaths annually attributed to its effects. This has underscored the urgency for the implementation of effective prevention strategies. However, future climate change is likely to increase the frequency and duration of severe weather conditions, thereby promoting exposure to air pollution and exacerbating the impacts on human health [7]. A previous study indicated that tropical and subtropical populations of *Cx. tritaeniorhynchus* exhibited higher genetic diversity and that the effects of environmental factors (climatic conditions) provide significant advantages for the establishment and expansion of *Cx. tritaeniorhynchus* [8]. An understanding of the impact of environmental factors on JE can provide valuable insights into its morbidity and mortality, which remain unclear.

JE poses a significant public health threat, particularly in Asian countries, especially in South Asia, Southeast Asia, and East Asia [9]. Several countries in these regions have successfully implemented various intervention measures to reduce JE morbidity, including early diagnosis, prompt treatment, national immunization programs, and effective vector control strategies [10,11]. Despite these efforts, over 3 billion people still reside in JE-endemic countries, with an estimated 67,900 cases occurring annually [8]. The severity of JE is evident in its high mortality rate, with approximately 20%‐30% of cases resulting in fatalities. Additionally, 30%‐50% of JE survivors experience significant long-term neurological sequelae [9,12]. Previous studies have also highlighted the ongoing public health concern posed by JE in Taiwan [13].

Nestled at 23°4' north latitude and 121°0' east longitude, Taiwan enjoys a subtropical climate with average monthly temperatures ranging from a comfortable 16 to 29 °C. Average monthly relative humidity hovers between 75% and 90%. Despite its status as a developed nation with a per capita gross domestic product of US$35,244 [14], Taiwan continues to grapple with the challenges posed by JE. Despite occasional imported cases of JE in recent years [15], local infections persist in Taiwan, indicating limitations in the effectiveness of preventive measures in controlling or eradicating the disease. The number of JE cases in Taiwan ranges from sporadic confirmed cases to cluster epidemics, with the middle-aged population exhibiting the highest rate of JEV. Addressing the disease burden caused by JEV is imperative, and effective public health measures should be adopted in Taiwan. Although some studies provided epidemiological data on JE in Taiwan [16], their study did not delve into the clinical symptoms of JE patients over the past decade nor investigate the potential association between climate and air pollution factors and the number of cases [17]. The objective of this study is to conduct a comprehensive investigation of the epidemiological, clinical, and environmental characteristics of JE in Taiwan. To this end, population surveillance data gathered over a 13-year period will be used.

## Methods

### Study Design and Population

#### Study Area

Taiwan is an island in East Asia located between 21°45’ N and 25°56’ N. The Tropic of Cancer (23.5° N) passes through Chiayi City, situated in south-central Taiwan, dividing the entire island into 2 climate zones. Taiwan has a total land area of 35,980 km² and a population of approximately 23 million people, resulting in an average population density of 635 individuals per km². The northern part of Taiwan belongs to the subtropical climate zone, whereas the southern part belongs to the tropical climate zone. Consequently, the weather in Taiwan is relatively warm, with high humidity occurring throughout the year [18,19].

#### Case Definitions

A clinical case was defined as an individual of any age experiencing an acute onset of fever accompanied by a change in mental status and new onset of seizures (excluding simple febrile seizures) at any time of the year [9,13]. A confirmed case was defined as a clinical case with positive laboratory test results, including the presence of IgM antibodies specific to the JE virus in a single sample of cerebrospinal fluid (CSF) or serum; a 4-fold increase in IgG antibodies; the detection of JE virus antigens in tissue via immunohistochemistry; or the detection of the JE virus genome in serum, plasma, blood, CSF, or tissue samples. Additionally, cases meeting the clinical case definition and epidemiologically linked to a confirmed case were also considered confirmed [20,21].

### Data Collection

#### Surveillance for JE Infection

JE has been categorized as a notifiable infectious disease since 1955. Physicians are required to report all cases that meet the case definition of JE, collect samples, and send them to the Taiwan Centers for Disease Control (TCDC) within one week of the case being reported for examination [20]. We collected data from all JE-confirmed cases reported to the TCDC from January 2008 to December 2020. The reported information included patient age, sex, area of residence, geographic location, seasonal variation of exposure, and date of JE onset.

#### Laboratory Tests

In 1998, the TCDC developed an E/M-specific capture IgM and IgG enzyme-linked immunosorbent assay (E/M-specific IgM/IgG ELISA) for JE and dengue fever (DF) [13,22,23]. Hemagglutination inhibition (HI) and E/M-specific IgM/IgG ELISA are both employed as screening tools for detecting antibodies against JE and DF [24-26]. This study employed a previously described differential testing algorithm [26]. Briefly, positive samples on the JE ELISA underwent further testing using DF ELISA. The combined results from both assays informed the final interpretation. Positive JE ELISA and negative DF ELISA indicated the presence of JEV antibodies only, while positive results in both tests suggested a false-positive JE result. Long-term evaluation demonstrated high sensitivity and specificity of the E/M-specific IgM/IgG ELISA, effectively differentiating JEV infections from dengue virus infections [26]. Since 2001, real-time polymerase chain reaction (PCR) analysis of acute-phase serum samples collected within 7 days of symptom onset and CSF from individuals diagnosed with JE has been employed for diagnostic purposes [27]. Nevertheless, enzyme-linked immunosorbent assay (ELISA) remains the primary diagnostic testing method, with the E/M-specific IgM/IgG ELISA being the most widely used ELISA method.

The JE laboratory has confirmed that clinical cases meeting one of the following specific laboratory criteria should be defined as confirmed cases of JE: (1) an HI titer of the convalescent serum of 1:160 and at least a 4-fold increase between the HI titers of convalescent and acute sera [24,25]; (2) an HI titer of 1:320 derived from a single serum sample [24,25]; (3) an IgM-positive serum sample, as determined by an ELISA test, or a fourfold increase in IgG levels between paired serum samples [25]; (4) a sample exhibiting a positive real-time PCR [25]; (5) a sample that has been found to be positive for indirect immunofluorescence antibody staining following the isolation of the virus from CSF [25,28]; (6) virus isolation can be accomplished through cell culture techniques using either the mosquito C6/36 cell line or plaque assays with the BHK-21 cell line [26]; and (7) an alternative approach is the extraction of viral RNA from JEV-infected culture medium using the QIAamp Viral RNA Mini Kit (Qiagen, Germantown, MD) [15].

### Surveillance of Environmental Factors

The dataset comprised monthly air pollutant data from 2008 to 2020, obtained from the air quality monitoring network of the Environmental Protection Agency. The pollutants analyzed included total suspended particulates, particulate matter (PM) 2.5 and 10 (PM2.5 and PM10), nitrogen dioxide (NO_2_), sulfur dioxide (SO_2_), carbon monoxide (CO), ozone (O_3_), dust fall volume, and lead concentration [29]. Furthermore, monthly weather data from the Meteorological Bureau of the Ministry of Communications for the same period were analyzed. The meteorological variables included temperature, rainfall, relative humidity, atmospheric pressure, the number of days with precipitation, and the duration of sunshine [30]. Statistical analyses and correlation tests were employed to assess the temporal and spatial trends in air pollutants and meteorological factors, as well as their potential association with JE case numbers. The data collected for this study were stored in the supplementary file.

### Statistical Analysis

This study presents a retrospective historical analysis of all cases of JE diagnosed in Japan since 2008, including both domestic and imported cases. The number of individuals diagnosed with JE from 2008 to 2020 was confirmed, and the epidemiological characteristics of these cases were examined. These characteristics included sex (male and female), age groups (<20, 20‐39, 40‐59, and ≥60 years old), time of diagnosis (spring, summer, fall, and winter), and area of residence (northern, central, southern, and eastern Taiwan). Disparities and outcomes were analyzed. Our analysis then focused on the variables of sex, age, time of diagnosis, changes in living area, trends, and related outcomes for cases of JE from 2011 to 2020. Descriptive data are presented as means and summaries where appropriate. Categorical variables were compared using chi-squared tests. Odds ratios were calculated using logistic regression, with 95% confidence intervals estimated using parameter estimation. All statistical analyses were performed using SPSS (IBM SPSS version 21; Asia Analytics Taiwan Ltd). All statistical tests were 2-tailed, with an α value of .05. A *P* value less than .05 was considered statistically significant.

### Ethical Considerations

The ethical considerations of this study were consistent with those of Holland et al [31] in *JAMA Psychiatry* and Pan et al [32] in *BMC Infectious Diseases*. All case data are accessible via the internet, with information regarding JE cases available on the TCDC website. This study was deemed exempt from ethical approval according to the Communicable Disease Control Act of Taiwan [33] because the datasets used were deidentified and the study did not involve any of the datasets in a way that could potentially lead to the identification of individuals. Along with the absence of personal identifiers and the absence of any potential harm to individuals, the study was conducted in accordance with institutional research guidelines to ensure scientific rigor and ethical standards.

## Results

### Demographic Data

**Figure 1.**
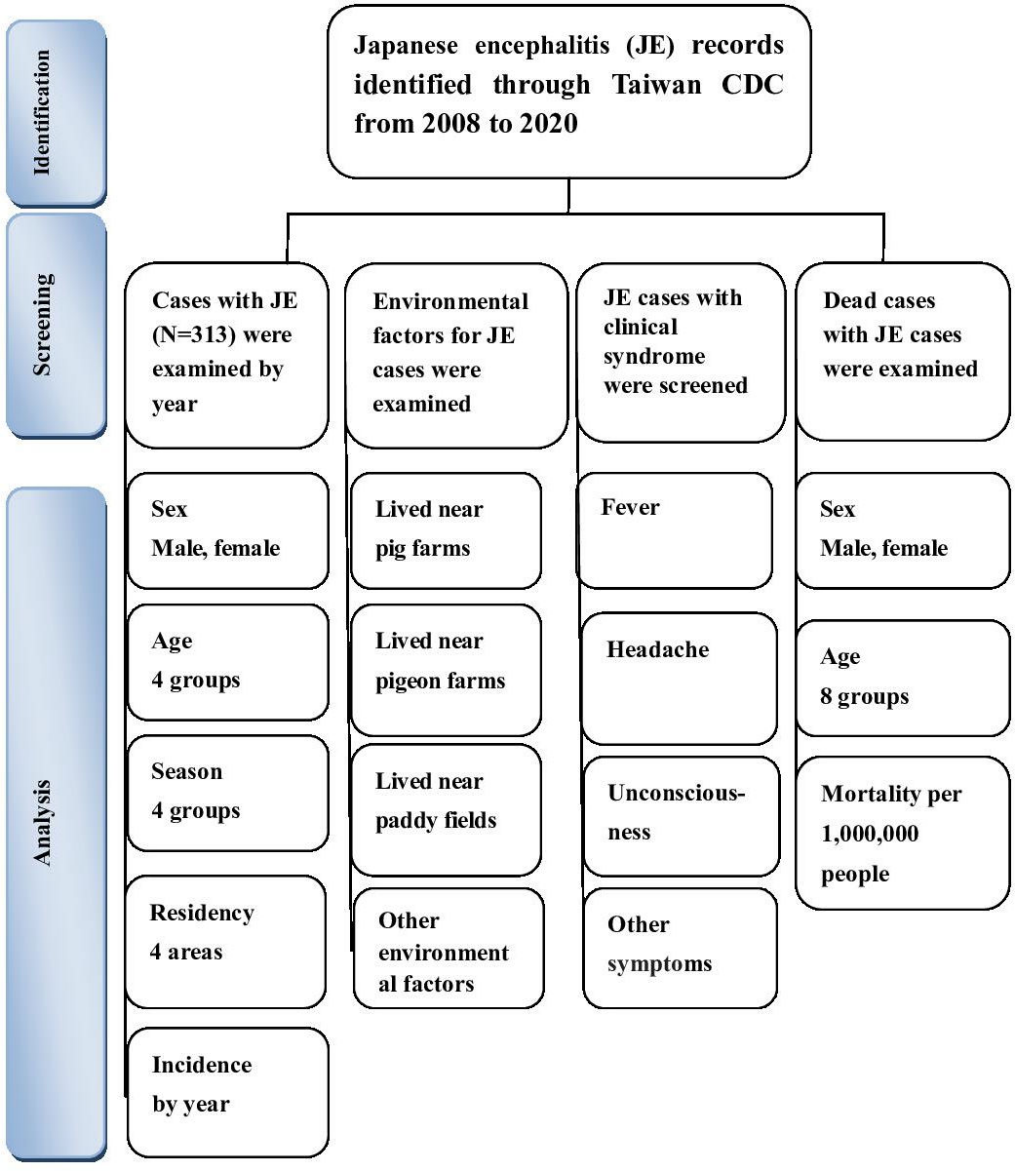
Flowchart of the study sample selection from the Taiwan Centers for Disease Control Database (CDC) in Taiwan from January 2008 to December 2020.

The figures and tables selected for analysis in this study are representative of an examination of demographic characteristics (incidence rates), patient clinical symptoms, environmental factors, air pollution, and climate factors on JE in Taiwan over the recent 13-year period. The flowchart of this study is shown in Figure 1. There were 313 confirmed cases of local and imported infection, consisting of 309 (98.7%) confirmed local cases, 195 (62.3%) male patients, 182 (58.1%) patients aged 40‐59 years, 245 (78.3%) cases in summer, and 145 (46.3%) cases in the southern region. There were 4 (1.3%) confirmed cases of imported infection (Table 1). There was no statistically significant difference between the number of confirmed cases in 2008 and 2020 and the relationship between different identities (local or imported), sex, age group, season, and place of residence (all *P*>.05) (Table 1). There was a statistically significant difference between the number of confirmed cases in 2008 and 2020 and the relationship between different seasons and place of residence (all *P*<.05) (Table 1). The main environmental factors associated with JE were paddy fields (160/582, 27.5%), pig farms (158/582, 27.1%), pigeon farms (125/582, 21.5%), poultry farms (65/582, 11.2%), ponds (29/582, 5%), and domestic pets (18/582, 3.1%), as indicated in Table 2.

**Table 1.** Epidemiological features of Japanese encephalitis cases in Taiwan from 2008 to 2020.

Variable	Total	Year													*P* value
Domestic		2008 (n=17)	2009 (n=18)	2010 (n=33)	2011 (n=22)	2012 (n=32)	2013 (n=16)	2014 (n=18)	2015 (n=30)	2016 (n=23)	2017 (n=25)	2018 (n=37)	2019 (n=21)	2020 (n=21)	
Individual, n															.12
Domestic	309	17	17	32	22	32	16	18	30	23	25	37	19	21	
Imported	4	0	1	1	0	0	0	0	0	0	0	0	2	0	
Sex, n															.90
Male	195	12	13	22	14	17	12	12	19	12	15	20	13	14	
Female	118	5	5	11	8	15	4	6	11	11	10	17	8	7	
Age group, n															.26
<20	10	3	1	1	1	0	1	0	0	1	0	1	0	1	
20‐39	68	5	3	11	8	9	5	3	7	2	4	6	3	2	
40‐59	182	9	9	16	9	17	9	10	20	16	14	24	14	15	
≥60	53	0	5	5	4	6	1	5	3	4	7	6	4	3	
Season, n															<.001
Spring	40	1	2	2	0	10	2	0	0	2	3	10	7	1	
Summer	245	15	14	29	20	19	14	15	28	14	19	25	13	20	
Fall	28	1	2	2	2	3	0	3	2	7	3	2	1	0	
Winter	0	0	0	0	0	0	0	0	0	0	0	0	0	0	
Residency, n															.008
Northern	78	2	5	10	9	4	5	2	7	4	8	10	5	7	
Central	65	4	2	6	5	5	3	5	4	5	5	9	5	7	
Southern	145	9	4	14	4	21	8	11	18	11	10	17	11	7	
Eastern	25	2	7	3	4	2	0	0	1	3	2	1	0	0	

**Table 2.** Environmental factors for Japanese encephalitis cases in Taiwan between 2008 and 2020.

Variable (lived nearby)		Year												
	Total, n	2008, n	2009, n	2010, n	2011, n	2012, n	2013, n	2014, n	2015, n	2016, n	2017, n	2018, n	2019, n	2020, n
Pig farms	158	4	6	8	10	23	10	11	13	11	13	25	13	11
Pigeon farms	125	7	4	14	2	17	5	7	9	11	10	19	8	12
Paddy fields	160	9	6	17	9	16	9	11	14	11	8	26	14	10
Egret nests	6	1	—^a^	1	—	4	—	—	—	—	—	—	—	—
Henhouses	2	1	1	—	—	—	—	—	—	—	—	—	—	—
Pond	29	2	2	3	1	7	3	2	3	1	1	3	1	—
Clam breeding farms	1	—	1	—	—	—	—	—	—	—	—	—	—	—
Poultry farms	65	—	—	9	6	11	3	—	5	6	5	12	4	4
Goat farms	4	—	—	2	—	1	—	—	—	—	1	—	—	—
Domestic pets	18	—	—	1	5	5	3	—	2	1	—	—	—	1
Farmlands	6	—	—	—	3	3	—	—	—	—	—	—	—	—
Orchards	8	—	—	—	3	5	—	—	—	—	—	—	—	—

a Not applicable

### Clinical Symptoms

The main clinical symptoms of JE were fever (280/866, 32.3%), unconsciousness (162/866, 18.7%), headache (136/866, 15.7%), stiff neck (63/866, 7.3%), psychiatric symptoms (59/866, 6.8%), and vomiting (49/866, 5.7%), as shown in Table 3.

There were 24 deaths from JE, of which 15 (62.5%) were male and 9 (37.5%) were aged 50‐60 years. The mortality rate (per 1,000,000 people) ranged from 0.04 to 0.17 between 2008 and 2020 (Table 4).

**Table 3.** Clinical symptoms of Japanese encephalitis cases in Taiwan from 2008 to 2020.

Variable		Year												
Total	2008	2009	2010	2011	2012	2013	2014	2015	2016	2017	2018	2019	2020
Fever, n	280	16	18	30	18	26	15	14	28	21	21	33	20	20
Headache, n	136	13	5	19	13	15	8	7	9	9	8	14	6	10
Stiff necks, n	63	1	6	3	6	6	3	5	3	5	7	11	3	4
Muscle cramps, n	35	7	2	5	—^a^	—	3	2	6	2	3	2	—	3
Vomiting, n	49	4	2	11	4	5	3	1	2	1	3	8	4	1
Speaking difficulty, n	9	—	2	—	3	4	—	—	—	—	—	—	—	—
Coma, n	30	4	5	12	7	2	—	—	—	—	—	—	—	—
Unconscious, n	162	5	6	21	11	9	11	9	13	17	14	20	12	14
Encephalitis, n	3	—	—	—	1	2	—	—	—	—	—	—	—	—
Paralysis, n	1	—	—	—	1	—	—	—	—	—	—	—	—	—
Psychological symptoms, n	59	—	—	—	—	9	—	5	11	5	5	15	6	3
Paralyzed limbs, n	3	—	—	—	—	3	—	—	—	—	—	—	—	—
Diarrhea, n	2	—	—	—	—	2	—	—	—	—	—	—	—	—
Muscular ache, n	2	—	—	—	—	2	—	—	—	—	—	—	—	—
Encephalitic stimulation symptoms, n	10	—	—	—	—	—	—	1	3	2	1	2	—	1
Dystonia, n	16	—	—	—	—	—	—	—	4	4	2	1	2	3
Meningitis symptoms, n	6	—	—	—	—	—	—	—	—	—	—	—	—	3
Aseptic meningitis, n	—	—	—	—	—	—	—	—	—	—	—	—	—	3

a Not applicable.

**Table 4.** The mortality rate per 1,000,000 people and the characteristics of deceased cases with Japanese encephalitis from 2008 to 2020^a^ in Taiwan.

Variable	2008 (n=2)	2009 (n=2)	2010 (n=2)	2011 (n=1)	2013 (n=1)	2014 (n=3)	2015 (n=1)	2016 (n=4)	2017 (n=3)	2018 (n=2)	2019 (n=3)
Mortality rate per 1,000,000 people	0.09	0.09	0.09	0.04	0.04	0.13	0.04	0.17	0.13	0.08	0.13
Sex, n											
Male	1	2	0	1	0	3	0	3	2	2	1
Female	1	0	2	0	1	0	1	1	1	0	2
Age group, n											
<20	—^b^	—	—	—	—	—	—	—	—	—	—
20‐39	—	—	1	—	—	1	—	—	—	—	—
40‐59	1	1	1	—	1	2	1	3	1	—	3
≥60	1	1	—	1	—	—	—	1	2	2	—

a There were no reported deaths due to Japanese encephalitis in 2012 and 2020.

b Not applicable

### Regression Analysis

Linear regression analysis showed that air pollution factors were associated with JE cases: SO_2_ concentration (*β*=2.184, SE 0.922; *P*=.02) and O_3_ concentration (*β*=−0.157, SE 0.063; *P*=.01) (Table 5). Linear regression analysis showed that climatic factors were associated with JE cases (R^2^=0.344, *F_6,137_*=11.980, *P*<.001) (Table 6).

**Figure 2.**
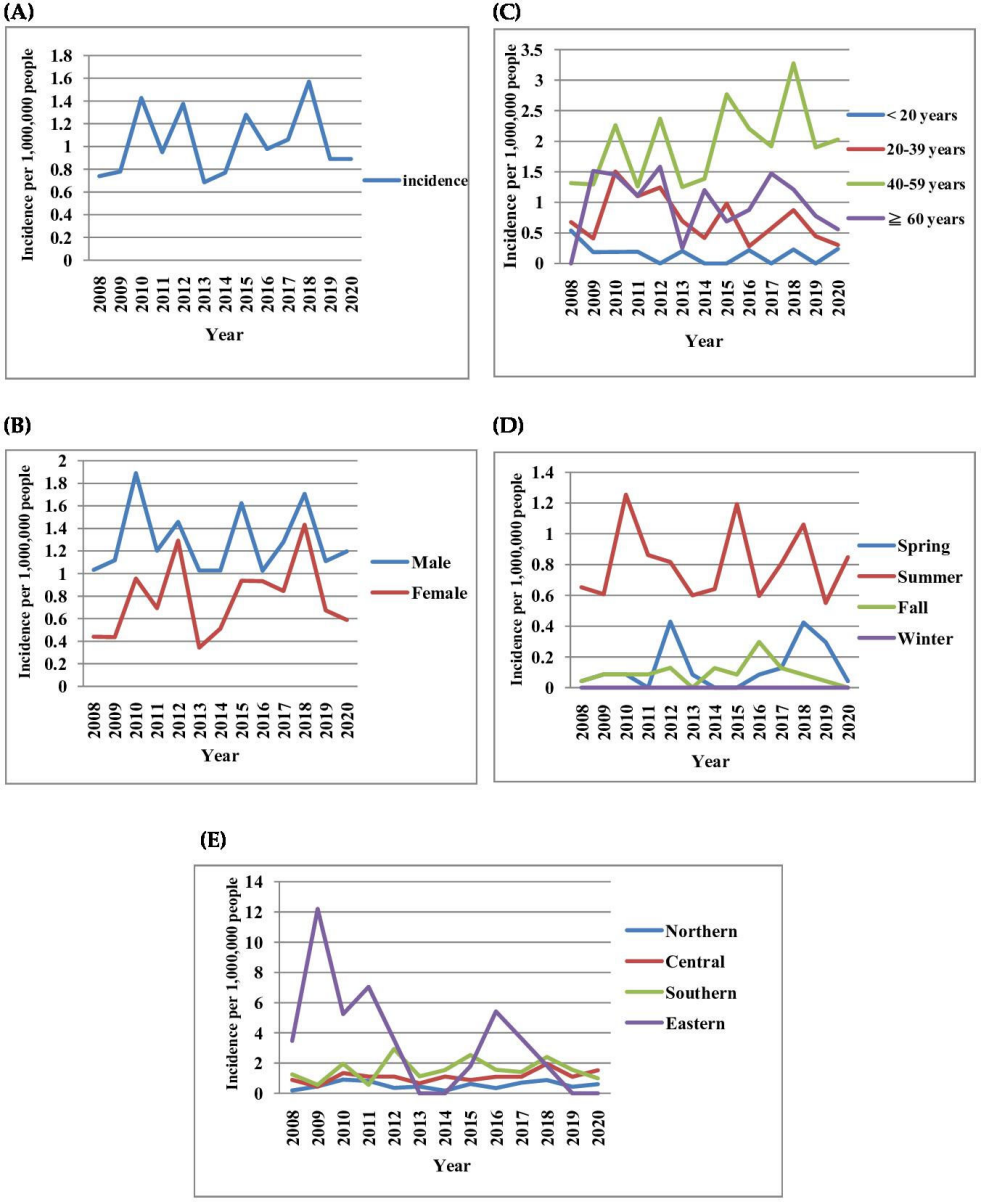
Incidence of confirmed JEV among patients in Taiwan according to (**A**) population, (**B**) gender, (**C**) age, (**D**) season, and (**E**) region of residence by year from 2008 to 2020.

**Figure 3.**
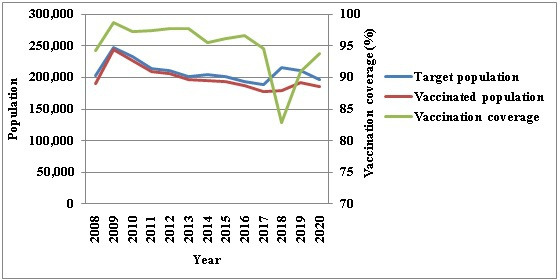
National target population, vaccinated population, and vaccination coverage for Japanese encephalitis in Taiwan from 2008 to 2020.

The incidence of confirmed cases of JE per million people was 0.69‐1.57 (Figure 2A); 1.02‐1.89 in males (Figure 2B); 1.25‐3.27 in the 40‐59 years age group (Figure 2C); 0.55‐1.25 in summer (Figure 2D); and 0‐12.2 in the northern region (Figure 2E), during 2008‐2020. The national coverage of JE vaccine in Taiwan is shown in Figure 3.

**Table 5.** Association between air pollution factors and Japanese encephalitis cases examined through multiple linear regression analysis. *R*^2^=0.417; *F* test (*df*) –> 6.824 (9, 86); *P* <.001; n=96.

Variables	Nonstandardization coefficient		*P* value
*β* value	Standard error
TSP^a^ (μg/m^3^)	−0.028	0.055	.61
PM^b^ 2.5 (μg/m^3^)	0.022	0.177	.90
PM 10 (μg/m^3^)	0.007	0.124	.95
SO_2_ (ppb)	2.184	0.922	.02
CO (ppm)	−17.244	19.092	.37
NO_2_ (ppb)	−0.342	0.564	.55
O_3_ (ppb)	−0.157	0.063	.01
Dustfall volume(tonne/km^2^/month)	0.029	0.509	.96
Lead (μg/m^3^)	43.751	72.026	.55

a TSP: total suspended particulate.

b PM: particulate matter.

**Table 6.** Relationship between climate factors and Japanese encephalitis cases analyzed using multiple linear regression analysis. *R*^2^=0.344; *F* test (*df*) –> 11.980 (6, 137); *P*<.001; n=144.

Variables	Nonstandardization coefficient		*P* value
*β* value	Standard error
Temperature (℃)	−0.025	0.192	.90
Precipitation (mm)	−0.004	0.004	.30
Relative humidity (%)	0.380	0.163	.02
Mean pressure (hPa)	−0.247	0.155	.12
Number of days with precipitation ≥0.1 mm (day)	−0.017	0.199	.93
Sunshine duration (h)	0.029	0.016	.08

## Discussion

### Principal Findings

JE is the most prevalent cause of viral encephalitis in Asia, with transmission occurring through mosquito bites. It is endemic in most of South and Southeast Asia, but the number of cases can vary significantly between regions [34]. The study yielded several significant findings. With regard to epidemiological characteristics, the data indicated that men, middle-aged individuals, those residing in summer, and residents of southern Taiwan are at greater risk. The primary clinical symptoms observed were fever, headache, and unconsciousness. An increase in the concentration of the air pollutant SO₂ and the relative humidity percentage, when considered as climatic factors, was found to correlate with an increase in the number of JE cases.

Epidemiology examines the epidemiological characteristics associated with disease transmission, presentation, and outcome. This discipline has consistently been influenced by policy interventions and disease prevention measures [35,36]. From 2008 to 2020, a total of 313 cases of JE were confirmed in Taiwan. Of these cases, 1.3% were imported, while 98.7% were indigenous, indicating that JE is endemic in Taiwan. The high incidence rate among men is consistent with previous studies [37-39]. It can be postulated that the underlying cause may be differences in personal hygiene habits. Furthermore, most cases occur in individuals aged 40-59 years. Patients in this age group exhibited a higher incidence compared to those younger than 20 years (1.94 vs 0.73), which is consistent with previous studies [13,40,41]. The government of Taiwan can use the similarities and differences in epidemiological characteristics as a foundation for developing and implementing its epidemic prevention policy or strategy.

Among all epidemiological characteristics, seasonal variation is the most susceptible to the distribution of all confirmed cases. In essence, seasonal variation is sufficient to affect the significant increase or decrease of confirmed cases, thereby indicating that the disease in South Asia, Southeast Asian countries, or even Taiwan is endemic [42]. The disease in question typically manifests during the summer months in Taiwan, as evidenced by the findings of previous studies [43-46]. According to the Central Weather Administration of Taiwan, the average summer temperature in Taiwan is 26.4 °C [47]. Consequently, the findings indicate that temperature is a pivotal factor influencing the vector competence and survival of infected mosquitoes. Moreover, southern Taiwan is situated in rural and suburban areas. A previous study indicated that in rural and suburban areas, traditional rice farming and intensive pig breeding provide an ideal environment for both mosquito development and the transmission of JEV among humans. Our findings align with those of previous studies [48-50]. In other words, there are always traces of the JEV in Taiwan that present a threat to public health and increase the clinical medical burden. At this juncture, it is imperative to address these significant challenges head-on, employing strategies to prevent and control them. It is imperative that the Taiwanese government’s health department proactively propose local prevention strategies and implement effective surveillance operations to control the epidemic and reduce the number of cases, thereby eliminating the health threat.

JEV is spread by mosquitoes within an enzootic cycle that includes reservoirs among pigs and birds, with humans serving as unintentional dead-end hosts. In the past decade, both serological and genetic evidence from previous studies suggest the presence of JEV in the local fauna [51]. As in the previous study, workplaces of cases in Taiwan are commonly found in pig farms, pigeon farms, and paddy fields [52,53]. This study recommends that Taiwan’s government health departments should increase publicity about measures to prevent JE by raising pigs and pigeons in rural areas.

A previous study demonstrated that while the impacts of the COVID-19 lockdown on various sectors, including the economy, research, travel, education, and sports, were clear, the effects on the occurrence of vector-borne diseases (VBDs) were less apparent [54]. The number of cases reported was compared with the number of cases predicted for each year from 2015 to 2020 for the VBDs (JE) under study to infer whether the COVID-19 lockdown had any impact on their prevalence in Taiwan. The actual number of cases was 21, both before and after the lockdown (2019 and 2020). The predicted number of cases for 2020, based on the trend of the previous 5 years (2015‐2019), was also close to the actual number of cases. This suggests that the JEV has been consistently present in the local area of Taiwan for a long time. During the COVID-19 period, the number of JE cases did not fluctuate significantly. The analysis suggests that the lockdown did not have a significant impact on the incidence of VBDs such as JE.

In the majority of cases, patients infected with JE do not exhibit any clinical symptoms [55,56]. Consequently, the clinical symptoms observed in JE cases in Taiwan are consistent with those previously documented in other studies [57,58]. It is challenging to differentiate between JEV and other pathogens that can cause encephalitis and meningitis, including enterovirus, mumps virus, mycoplasma, herpes virus, and other viruses. This necessitates the use of viral culture or serological examination [59]. Therefore, this study proposes that the current understanding of JE serum complex flavivirus cross-reactivity, which may result in variable clinical outcomes, could inform future preventive and therapeutic interventions.

During the investigation period of this study, Taiwan exhibited a consistently low incidence rate of JE and its associated mortality from 2008 to 2020. The average incidence and mortality rates were 1.03 cases per 1,000,000 people and 0.09 cases per 1,000,000 people, respectively. A total of 10 cases of JE were reported in children. Consequently, the study indicates that the JE vaccination offered a moderate level of protection among children in JE-endemic Taiwan, a finding consistent with previous studies [60]. The implementation of the JE immunization program has been a pivotal factor in controlling the spread of JE. It is necessary to sustain a high vaccination coverage rate for JE and reinforce the disease surveillance system to ensure the efficacious control and eventual eradication of JE [61].

The prospective effects of climate change on public health are a rapidly expanding field of investigation. This encompasses the immediate consequences of more intense heat waves and declining food security, in addition to the indirect influences on the prevalence of infectious diseases [62-66]. Research has demonstrated that the proximity of collection sites to human dwellings (adjusted odds ratio [AOR] 2.02, *P*=.009) and a relative humidity exceeding 80% (AOR 2.40, *P*=.001) are significant independent risk factors for the transmission of JE viruses [67]. This finding is consistent with the present study. The results of this study indicate a positive correlation between relative humidity and JE cases (β=0.380, *P*=.02) through multiple linear regression analysis. The findings of this study indicate that climate change is a significant factor influencing the incidence of infectious diseases. It is therefore recommended that public health and epidemic prevention experts give this issue their attention, with the implementation of early planning measures to reduce the risk of disease.

Air pollution represents one of the most significant challenges of our era, affecting not only climate change but also public and individual health through increased morbidity and mortality [68]. A multitude of pollutants are significant contributors to human disease [69]. A previous study indicated that a comparison of the combined anthropogenic and environmental risk factors of major mosquito-borne diseases, specifically JE in Thailand, revealed that higher SO_2_ surface concentrations were negatively associated with disease case counts [70]. However, the results of the linear regression analysis indicated that there was a positive association between SO_2_ concentration and JE (β=2.184, *P*=.02). The concentration of O_3_ was found to be negatively correlated with JE (β=−0.157, *P*=.01). To the best of our knowledge, this is the first study to demonstrate that the number of cases of JE increased as the concentration of the air pollutant SO_2_ increased, and that there was a positive correlation. The study suggests that the high concentration of SO_2_ in summer, which is a season prone to JE, may be a contributing factor. It was hypothesized that there was a correlation between these air pollutants and the disease. Consequently, this study proposes that Taiwan’s official policy should implement a rigorous monitoring program to assess local climate factors and air pollution concentration changes. In the event of minor fluctuations or significant fluctuations in environmental data, it is imperative that the media and the public be promptly informed. This will enable individuals to take prompt action to mitigate environmental pressures and the threat to public health.

### Limitations

One limitation of the present study is that the data provided by the Taiwan National Infectious Disease Statistics System lack information about the genotypes or strains of the JEV isolated. Consequently, the type of JEV strain that spread to Taiwan and the affinity between virus strains in Taiwan and other countries were not analyzed in this study. Nevertheless, the advantage of this study was the access to the diverse data provided by Taiwan’s public sector on its web-based platform (including the initial version of the platform) and the evaluation of the impact of the COVID-19 pandemic on the epidemiological features of typhoid and paratyphoid. The information from existing public network platforms in Taiwan is both timely and accurate.

### Conclusions

This study is the first to report on the epidemiological characteristics, clinical symptomatology, climatic factors, and air pollutants associated with JE cases in Taiwan between 2008 and 2020. In 2018, the incidence rate of JE was the highest (1.57 per million people). In recent years, the risk of local cases has increased at a rapid pace, resulting in a significant burden of disease, public health challenges, and epidemic prevention. The distribution of JE in Taiwan is regional in nature. The risk of JE among patients residing in different areas increases with age. The clinical manifestations of JE are variable, and the primary areas of endemicity are pig farms, pigeon farms, and paddy fields. The JE vaccine coverage rate in Taiwan is high, with an average of 94.9%. The concentration of certain environmental factors (SO_2_, O_3_) may influence the increase or decrease in JE cases. The identification of crucial data will facilitate future surveillance and research activities in Taiwan.

## Abbreviations

AOR: adjusted odds ratio
CSF: cerebrospinal fluid
DF: dengue fever
HI: hemagglutination inhibition
JE: Japanese encephalitis
JEV: Japanese encephalitis virus
PM: particulate matter
TCDC: Taiwan Centers for Diseases Control
VBD: vector-borne disease

## References

[1] (2022). Key facts of japanese encephalitis. WHO.

[2] (2022). Japanese encephalitis: symptoms, diagnosis, and treatment. Centers for Disease Control and Prevention.

[3] Wilder-Smith A, Halstead SB (2010). Japanese encephalitis: update on vaccines and vaccine recommendations. Curr Opin Infect Dis.

[4] Leinikki P, Zuckerman AJ, Banatvala JE, Pattison JR (2004). Principles and Practice of Clinical Virology.

[5] Lindenbach B, Rice C, Fields BN, Howley PM, Griffin DE, Lamb RA, Martin MA, Roizman B, Straus SE (2001). Fields Virology.

[6] Ghosh D, Basu A (2009). Japanese encephalitis-a pathological and clinical perspective. PLoS Negl Trop Dis.

[7] Hong C, Zhang Q, Zhang Y (2019). Impacts of climate change on future air quality and human health in China. Proc Natl Acad Sci USA.

[8] Zhang Y, Wang H, Du J (2024). Population genetic structure of *Culex tritaeniorhynchus* in different types of climatic zones in China. BMC Genomics.

[9] Centers for Disease Control and Prevention (CDC) (2013). Japanese encephalitis surveillance and immunization—Asia and Western pacific, 2012. MMWR Morb Mortal Wkly Rep.

[10] Heffelfinger JD, Li X, Batmunkh N (2017). Japanese encephalitis surveillance and immunization—Asia and Western Pacific Regions, 2016. Wkly Epidemiol Rep.

[11] Le Flohic G, Porphyre V, Barbazan P, Gonzalez JP (2013). Review of climate, landscape, and viral genetics as drivers of the Japanese encephalitis virus ecology. PLoS Negl Trop Dis.

[12] Fischer M, Hills S, Staples E, Johnson B, Yaich M, Solomon T, Scheld WM, Hammer SM, Hughes JM (2008). Emerging Infections 8.

[13] Chang YK, Chang HL, Wu HS, Chen KT (2017). Epidemiological features of Japanese encephalitis in Taiwan from 2000 to 2014. Am J Trop Med Hyg.

[14] (2022). Average GDP per person (USD) [Article in Mandarin]. National Statistics, ROC (Taiwan).

[15] Su CL, Yang CF, Teng HJ (2014). Molecular epidemiology of Japanese encephalitis virus in mosquitoes in Taiwan during 2005-2012. PLoS Negl Trop Dis.

[16] Wu YH, Chen JM, Su CY (2017). Occurrence of old age Japanese encephalitis: current situation in Taiwan. J Formos Med Assoc.

[17] Hsu JY, Hung CC, Tsou TP, Chen WC (2023). Epidemiology and risk factors of Japanese encephalitis in Taiwan, 2010-2022. PLoS Negl Trop Dis.

[18] Lin CL, Chang HL, Lin CY, Chen KT (2017). Seasonal patterns of Japanese encephalitis and associated meteorological factors in Taiwan. Int J Environ Res Public Health.

[19] Ho YC, Su BH, Su HJ (2015). The association between the incidence of mumps and meteorological parameters in Taiwan. Hum Vaccin Immunother.

[20] Taiwan National Infectious Disease Statistics System [Website in Mandarin].

[21] Solomon T, Thao TT, Lewthwaite P (2008). A cohort study to assess the new WHO Japanese encephalitis surveillance standards. Bull World Health Organ.

[22] Hsu LC, Chen YJ, Hsu FK (2014). The incidence of Japanese encephalitis in Taiwan--a population-based study. PLoS Negl Trop Dis.

[23] Ravi V, Robinson JS, Russell BJ (2009). Evaluation of IgM antibody capture enzyme-linked immunosorbent assay kits for detection of IgM against Japanese encephalitis virus in cerebrospinal fluid samples. Am J Trop Med Hyg.

[24] Clark CH, Casals J (1958). Techniques for hemagglutination inhibition with arthropod viruses. Am J Trop Med Hyg.

[25] (2007). Manual for the Laboratory Diagnosis of Japanese Encephalitis Virus Infection.

[26] Johnson BW, Goodman CH, Jee Y, Featherstone DA (2016). Differential diagnosis of Japanese encephalitis virus infections with the Inbios JE Detect™ and DEN Detect™ MAC-ELISA Kits. Am Soc Trop Med Hyg.

[27] Sapkal GN, Wairagkar NS, Ayachit VM, Bondre VP, Gore MM (2007). Detection and isolation of Japanese encephalitis virus from blood clots collected during the acute phase of infection. Am J Trop Med Hyg.

[28] Mathur A, Kumar R, Sharma S, Kulshreshtha R, Kumar A, Chaturvedi UC (1990). Rapid diagnosis of Japanese encephalitis by immunofluorescent examination of cerebrospinal fluid. Indian J Med Res.

[29] Environmental statistics inquiry network [Article in Mandarin]. Ministry of Environment.

[30] Monthly weather [Article in Mandarin]. Central Weather Administration.

[31] Holland KM, Jones C, Vivolo-Kantor AM (2021). Trends in US Emergency Department visits for mental health, overdose, and violence outcomes before and during the COVID-19 pandemic. JAMA Psychiatry.

[32] Pan CY, Liu WL, Su MP (2020). Epidemiological analysis of the Kaohsiung city strategy for dengue fever quarantine and epidemic prevention. BMC Infect Dis.

[33] (2023). Communicable Disease Control Act. Laws & Regulations Database of The Republic of China (Taiwan).

[34] Lopalco PL, Biasio LR (2024). Japanese encephalitis can be devastating. Ann Ig.

[35] Gordis L, Gordis L (2004). Epidemiology.

[36] Stanley SL (2003). Amoebiasis. Lancet.

[37] Srivastava N, Deval H, Mittal M (2022). Extent of disability among paediatric Japanese encephalitis survivors and predictors of poor outcome: a retrospective cohort study in North India. BMJ Open.

[38] Pommier JD, Gorman C, Crabol Y (2022). Childhood encephalitis in the Greater Mekong region (the SouthEast Asia Encephalitis Project): a multicentre prospective study. Lancet Glob Health.

[39] Udeze AO, Odebisi-Omokanye MB (2022). Sero-evidence of silent Japanese encephalitis virus infection among inhabitants of Ilorin, North-central Nigeria: a call for active surveillance. J Immunoassay Immunochem.

[40] Wu D, Chen X, Liu W (2021). Emergence of Japanese encephalitis among adults 40 years of age or older in northern China: epidemiological and clinical characteristics. Transbound Emerg Dis.

[41] Kwak BO, Hong YJ, Kim DH (2022). Changes in age-specific seroprevalence of Japanese encephalitis virus and impact of Japanese encephalitis vaccine in Korea. Clin Exp Pediatr.

[42] Lin FH, Chen BC, Chou YC (2022). The epidemiology of *Entamoeba histolytica* infection and its associated risk factors among domestic and imported patients in Taiwan during the 2011-2020 period. Medicina (Kaunas).

[43] Lee PI, Huang YC, Hwang KP (2020). Recommendations for the use of Japanese encephalitis vaccines. Pediatr Neonatol.

[44] Griffith MM, Fukusumi M, Kobayashi Y (2018). Epidemiology of vaccine-preventable diseases in Japan: considerations for pre-travel advice for the 2019 Rugby World Cup and 2020 Summer Olympic and Paralympic Games. Western Pac Surveill Response J.

[45] Folly AJ, Dorey-Robinson D, Hernández-Triana LM (2021). Temperate conditions restrict Japanese encephalitis virus infection to the mid-gut and prevents systemic dissemination in *Culex pipiens* mosquitoes. Sci Rep.

[46] Lo SH, Tang HJ, Lee SSJ (2019). Determining the clinical characteristics and prognostic factors for the outcomes of Japanese encephalitis in adults: a multicenter study from southern Taiwan. J Microbiol Immunol Infect.

[47] Monthly average climate [Article in Mandarin]. Central Weather Administration.

[48] Tian HY, Bi P, Cazelles B (2015). How environmental conditions impact mosquito ecology and Japanese encephalitis: an eco-epidemiological approach. Environ Int.

[49] Di Francesco J, Choeung R, Peng B (2018). Comparison of the dynamics of Japanese encephalitis virus circulation in sentinel pigs between a rural and a peri-urban setting in Cambodia. PLoS Negl Trop Dis.

[50] Lindahl JF, Ståhl K, Chirico J, Boqvist S, Thu HTV, Magnusson U (2013). Circulation of Japanese encephalitis virus in pigs and mosquito vectors within Can Tho city, Vietnam. PLoS Negl Trop Dis.

[51] Lim MJ, Loh ZY, Yeo HL (2022). Isolation and genetic characterization of Japanese encephalitis virus two decades after its elimination in Singapore. Viruses.

[52] Williams CR, Webb CE, Higgs S, van den Hurk AF (2022). Japanese encephalitis virus emergence in Australia: public health importance and implications for future surveillance. Vector Borne Zoonotic Dis.

[53] Walsh MG, Pattanaik A, Vyas N (2022). High-risk landscapes of Japanese encephalitis virus outbreaks in India converge on wetlands, rain-fed agriculture, wild Ardeidae, and domestic pigs and chickens. Int J Epidemiol.

[54] Mayilsamy M, Vijayakumar A, Veeramanoharan R, Rajaiah P, Balakrishnan V, Kumar A (2023). Impact of COVID-19 lockdown during 2020 on the occurrence of vector-borne diseases in India. J Vector Borne Dis.

[55] Sahu RC, Suthar T, Pathak A, Jain K (2022). Interventions for the prevention and treatment of Japanese encephalitis. Curr Infect Dis Rep.

[56] Turtle L, Solomon T (2018). Japanese encephalitis - the prospects for new treatments. Nat Rev Neurol.

[57] Pham D, Howard-Jones AR, Hueston L (2022). Emergence of Japanese encephalitis in Australia: a diagnostic perspective. Pathology (Phila).

[58] Guo H, Sun L, Shen X, Hu W (2022). A retrospective study of the clinical characteristics of Japanese encephalitis in adults. J Integr Neurosci.

[59] Wang LP, Yuan Y, Liu YL (2022). Etiological and epidemiological features of acute meningitis or encephalitis in China: a nationwide active surveillance study. Lancet Reg Health West Pac.

[60] Khare B, Kuhn RJ (2022). The Japanese encephalitis antigenic complex viruses: from structure to immunity. Viruses.

[61] Tandale BV, Khude PM, Deshmukh PS (2023). Effectiveness of Japanese encephalitis vaccination among children in central India. J Med Virol.

[62] Zhang WX, Zhao S, Pan C (2024). Mass immunisation to eradicate Japanese encephalitis: real-world evidence from Guizhou Province in 2005-2021. J Virus Erad.

[63] Mora C, Dousset B, Caldwell IR (2017). Global risk of deadly heat. Nat Clim Chang.

[64] Mazdiyasni O, AghaKouchak A, Davis SJ (2017). Increasing probability of mortality during Indian heat waves. Sci Adv.

[65] Schmidhuber J, Tubiello FN (2007). Global food security under climate change. Proc Natl Acad Sci USA.

[66] Springmann M, Mason-D’Croz D, Robinson S (2016). Global and regional health effects of future food production under climate change: a modelling study. Lancet.

[67] Wu X, Lu Y, Zhou S, Chen L, Xu B (2016). Impact of climate change on human infectious diseases: empirical evidence and human adaptation. Environ Int.

[68] Diptyanusa A, Herini ES, Indarjulianto S, Satoto TBT (2022). Estimation of Japanese encephalitis virus infection prevalence in mosquitoes and bats through nationwide sentinel surveillance in Indonesia. PLoS One.

[69] Manisalidis I, Stavropoulou E, Stavropoulos A, Bezirtzoglou E (2020). Environmental and health impacts of air pollution: a review. Front Public Health.

[70] Tewari P, Ma P, Gan G (2023). Non-linear associations between meteorological factors, ambient air pollutants and major mosquito-borne diseases in Thailand. PLoS Negl Trop Dis.

